# Methyl 3-(3-benzoyl­thio­ureido)propano­ate

**DOI:** 10.1107/S160053681100568X

**Published:** 2011-03-05

**Authors:** Ibrahim N. Hassan, Chong Yan Yi, Mohammad B. Kassim

**Affiliations:** a Fuel Cell Institute, Universiti Kebangsaan Malaysia, UKM 43600 Bangi, Selangor, Malaysia; b School of Chemical Sciences and Food Technology, Faculty of Science and Technology, Universiti Kebangsaan Malaysia, UKM 43600 Bangi, Selangor, Malaysia

## Abstract

In the title compound, C_12_H_14_N_2_O_3_S, the propyl acetate group and the benzoyl group adopt a *cis*–*trans* conformation, respectively, with respect to the thiono S atom across the C—N bonds. The phenyl ring is twisted relative to the the thio­urea mean plane, forming a dihedral angle of 24.16 (9)°. An intra­molecular N—H⋯O hydrogen bond occurs. The crystal packing is stabilized by inter­molecular N—H⋯O and C—H⋯O hydrogen bonds, forming a chain along the *a* axis.

## Related literature

For bond-length data, see: Allen *et al.* (1987 ▶). For related strutures, see: Yamin & Hassan (2004 ▶); Hassan *et al.* (2008*a* ▶,*b* ▶,*c* ▶, 2009 ▶), Hung *et al.* (2010 ▶). For a related synthesis, see: Hassan *et al.* (2008**a* ▶).* 
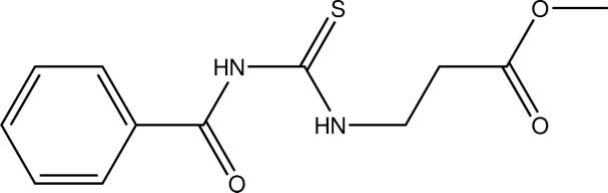


## Experimental

### 

#### Crystal data

C_12_H_14_N_2_O_3_S
*M* *_r_* = 266.31
Triclinic, 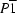

*a* = 7.5901 (18) Å
*b* = 8.2688 (19) Å
*c* = 10.547 (3) Å
α = 86.168 (5)°
β = 86.892 (4)°
γ = 81.545 (4)°
*V* = 652.6 (3) Å^3^
*Z* = 2
Mo *K*α radiation
μ = 0.25 mm^−1^
*T* = 298 K
0.35 × 0.31 × 0.23 mm

#### Data collection

Bruker SMART APEX CCD area-detector diffractometer
Absorption correction: multi-scan (*SADABS*; Sheldrick, 2000 ▶) *T*_min_ = 0.918, *T*_max_ = 0.945
8953 measured reflections
3229 independent reflections
2654 reflections with *I* > 2σ(*I*)
*R*_int_ = 0.024

#### Refinement

*R*[*F* ^2^ > 2σ(*F* ^2^)] = 0.057
*wR*(*F* ^2^) = 0.141
*S* = 1.12
3229 reflections
163 parameters
H-atom parameters constrained
Δρ_max_ = 0.27 e Å^−3^
Δρ_min_ = −0.22 e Å^−3^

### 

Data collection: *SMART* (Bruker, 2000 ▶); cell refinement: *SAINT* (Bruker, 2000 ▶); data reduction: *SAINT*; program(s) used to solve structure: *SHELXS97* (Sheldrick, 2008 ▶); program(s) used to refine structure: *SHELXL97* (Sheldrick, 2008 ▶); molecular graphics: *ORTEPIII* (Burnett & Johnson, 1996 ▶), *ORTEP-3 for Windows* (Farrugia, 1997 ▶) and *PLATON* (Spek, 2009 ▶); software used to prepare material for publication: *SHELXTL* (Sheldrick, 2008 ▶), *PARST* (Nardelli, 1995 ▶) and *PLATON*.

## Supplementary Material

Crystal structure: contains datablocks global, I. DOI: 10.1107/S160053681100568X/dn2657sup1.cif

Structure factors: contains datablocks I. DOI: 10.1107/S160053681100568X/dn2657Isup2.hkl

Additional supplementary materials:  crystallographic information; 3D view; checkCIF report

## Figures and Tables

**Table 1:** Hydrogen-bond geometry (Å, °)

*D*—H⋯*A*	*D*—H	H⋯*A*	*D*⋯*A*	*D*—H⋯*A*
N2—H2*A*⋯O1	0.86	1.94	2.625 (2)	136
N1—H1*A*⋯O2^i^	0.86	2.17	3.022 (2)	169
C1—H1*B*⋯O2^i^	0.93	2.50	3.187 (3)	130
C9—H9*A*⋯O1^ii^	0.97	2.59	3.464 (3)	150

Symmetry codes: (i) 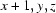; (ii) 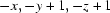.

**Fig. 1.**
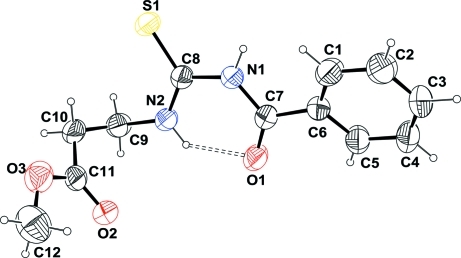
The molecular structure of (I), with the atom labeling scheme. Displacement ellipsoids are drawn at the 50% probability level. Hydrogen bond is shown as dashed line and H atoms are represented as small spheres of arbitrary radii.

**Fig. 2.**
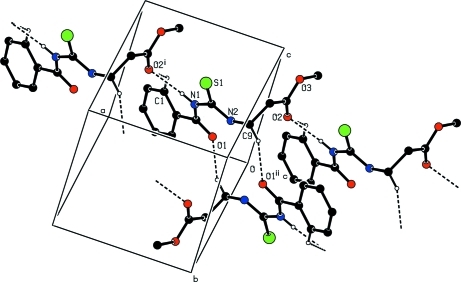
Partial packing view of (I) showing the chain formed by N-H···O and C-H···O hydrogen bonds which are shown as dashed lines. H atoms not involved in hydrogen bonding have been omitted for clarity. [Symmetry codes: (i) x+1, y, z; (ii) -x, -y+1, -z+1]

## supplementary crystallographic information

### Comment

The title compound, I, is a methyl ester derivative of beta-alanine thiourea analogous to our previous reported, methyl-2-(3-benzoylthioureido)acetate, II (Hassan *et al.* 2009).

The molecule maintains the same *cis*-*trans* conformation with respect to the positions of the methyl propanoate and benzoyl groups, relative to the S atom across the C—N bonds (Fig 1), respectively. The phenyl group, [C1/C2/C3/C4/C5/C6], and the methyl propanoate fragment, [O2/O3/C10/C11/C12], are essentially planar and the dihedral angle between them is 82.28 (11)°. The bond lengths (Allen *et al.*, 1987) and angles in the molecules are in normal ranges and comparable to those of II. The C=S bond length [1.670 (2) Å] is identical within experimental error to that of II [1.662 (5) Å]. The thiourea fragment,[S1/O1/N1/N2/C6/C7/C8/C9], is essentially planar with a maximum deviation of 0.037 (2) Å, at the atom N2. The phenyl ring [C1—C6] is inclined to the thiourea mean plane making a dihedral angle of 24.16 (9)° which is slightly larger than that of II [20.12 (19)°]. Atom O3 of the methyl propanoate group, (O2/O3/C10/C11/C12), has the maximum deviation 0.033 (2)Å from the mean plane.

There is one intramolecular hydrogen bonds, N2—H2A···O1 (Table 1) forming a pseudo-six-membered ring (N2/H2A/O1/C7/N1/C8). The intermolecular N1—H1A···O2, C1—H1B···O2 and C9—H9A···O1 hydrogen bonds, (Table 1), link the molecules into a chain parallel to the *a* axis (Fig 2).

### Experimental

The title compound was synthesized according to a previously reported compound (Hassan *et al.*, 2008*a*). A yellowish crystal, suitable for X-ray crystallography, was obtained by a slow evaporation from CH_2_Cl_2_ solution at room temperature (yield 79%).

### Refinement

H atoms of both C and N atoms were positioned geometrically and allowed to ride on their parent atoms, with *U*_iso_= 1.2*U*_eq_ (C) for aromatic 0.93 Å, *U*_iso_ = 1.2*U*_eq_ (C) for CH_2_ 0.97 Å, *U*_iso_ = 1.5*U*_eq_ (C) for CH_3_ 0.96 Å, *U*_iso_ = 1.2*U*_eq_ (N) for N—H 0.86 Å.

### Crystal data

**Table:**

C_12_H_14_N_2_O_3_S	*Z* = 2
*M**_r_* = 266.31	*F*(000) = 280
Triclinic, *P*1	*D*_x_ = 1.355 Mg m^−^^3^
Hall symbol: -P 1	Mo *K*α radiation, λ = 0.71073 Å
*a* = 7.5901 (18) Å	Cell parameters from 2861 reflections
*b* = 8.2688 (19) Å	θ = 1.9–28.3°
*c* = 10.547 (3) Å	µ = 0.25 mm^−^^1^
α = 86.168 (5)°	*T* = 298 K
β = 86.892 (4)°	Block, colourless
γ = 81.545 (4)°	0.35 × 0.31 × 0.23 mm
*V* = 652.6 (3) Å^3^	

### Data collection

**Table:**

Bruker SMART APEX CCD area-detector diffractometer	3229 independent reflections
Radiation source: fine-focus sealed tube	2654 reflections with *I* > 2σ(*I*)
graphite	*R*_int_ = 0.024
ω scans	θ_max_ = 28.3°, θ_min_ = 1.9°
Absorption correction: multi-scan (*SADABS*; Sheldrick, 2000)	*h* = −10→10
*T*_min_ = 0.918, *T*_max_ = 0.945	*k* = −11→11
8953 measured reflections	*l* = −14→14

### Refinement

**Table:**

Refinement on *F*^2^	Primary atom site location: structure-invariant direct methods
Least-squares matrix: full	Secondary atom site location: difference Fourier map
*R*[*F*^2^ > 2σ(*F*^2^)] = 0.057	Hydrogen site location: inferred from neighbouring sites
*wR*(*F*^2^) = 0.141	H-atom parameters constrained
*S* = 1.12	*w* = 1/[σ^2^(*F*_o_^2^) + (0.0624*P*)^2^ + 0.1657*P*] where *P* = (*F*_o_^2^ + 2*F*_c_^2^)/3
3229 reflections	(Δ/σ)_max_ < 0.001
163 parameters	Δρ_max_ = 0.27 e Å^−^^3^
0 restraints	Δρ_min_ = −0.22 e Å^−^^3^

### Special details

**Table:**

Geometry. All e.s.d.'s (except the e.s.d. in the dihedral angle between two l.s. planes) are estimated using the full covariance matrix. The cell e.s.d.'s are taken into account individually in the estimation of e.s.d.'s in distances, angles and torsion angles; correlations between e.s.d.'s in cell parameters are only used when they are defined by crystal symmetry. An approximate (isotropic) treatment of cell e.s.d.'s is used for estimating e.s.d.'s involving l.s. planes.
Refinement. Refinement of *F*^2^ against ALL reflections. The weighted *R*-factor *wR* and goodness of fit *S* are based on *F*^2^, conventional *R*-factors *R* are based on *F*, with *F* set to zero for negative *F*^2^. The threshold expression of *F*^2^ > σ(*F*^2^) is used only for calculating *R*-factors(gt) *etc*. and is not relevant to the choice of reflections for refinement. *R*-factors based on *F*^2^ are statistically about twice as large as those based on *F*, and *R*- factors based on ALL data will be even larger.

### Fractional atomic coordinates and isotropic or equivalent isotropic displacement parameters (Å^2^)

**Table:**

	*x*	*y*	*z*	*U*_iso_*/*U*_eq_	
S1	0.38506 (7)	0.35748 (7)	0.87738 (5)	0.05290 (19)	
O1	0.2339 (2)	0.3507 (2)	0.46757 (14)	0.0658 (5)	
O2	−0.1984 (2)	0.2418 (2)	0.71396 (14)	0.0594 (4)	
O3	−0.1813 (2)	0.1042 (2)	0.90333 (16)	0.0656 (5)	
N1	0.4213 (2)	0.3096 (2)	0.63147 (15)	0.0442 (4)	
H1A	0.5305	0.2778	0.6496	0.053*	
N2	0.1375 (2)	0.4127 (2)	0.70422 (15)	0.0472 (4)	
H2A	0.1086	0.4052	0.6273	0.057*	
C1	0.6740 (3)	0.1203 (3)	0.4582 (2)	0.0572 (6)	
H1B	0.6873	0.0919	0.5444	0.069*	
C2	0.7984 (3)	0.0513 (3)	0.3688 (3)	0.0685 (7)	
H2B	0.8948	−0.0242	0.3949	0.082*	
C3	0.7794 (3)	0.0944 (3)	0.2416 (2)	0.0644 (6)	
H3A	0.8635	0.0484	0.1820	0.077*	
C4	0.6379 (3)	0.2044 (3)	0.2022 (2)	0.0630 (6)	
H4A	0.6256	0.2331	0.1160	0.076*	
C5	0.5136 (3)	0.2726 (3)	0.2903 (2)	0.0544 (5)	
H5A	0.4173	0.3473	0.2630	0.065*	
C6	0.5295 (3)	0.2318 (2)	0.41872 (18)	0.0429 (4)	
C7	0.3820 (3)	0.3027 (3)	0.50660 (18)	0.0457 (5)	
C8	0.3050 (3)	0.3619 (2)	0.73260 (17)	0.0412 (4)	
C9	−0.0010 (3)	0.4804 (3)	0.7945 (2)	0.0524 (5)	
H9A	−0.0993	0.5396	0.7477	0.063*	
H9B	0.0463	0.5584	0.8432	0.063*	
C10	−0.0714 (3)	0.3532 (3)	0.88575 (19)	0.0548 (6)	
H10A	0.0264	0.2960	0.9341	0.066*	
H10B	−0.1579	0.4087	0.9453	0.066*	
C11	−0.1567 (2)	0.2306 (3)	0.82278 (19)	0.0498 (5)	
C12	−0.2732 (4)	−0.0183 (4)	0.8564 (3)	0.0828 (8)	
H12A	−0.2834	−0.1032	0.9219	0.124*	
H12B	−0.2074	−0.0642	0.7838	0.124*	
H12C	−0.3900	0.0309	0.8323	0.124*	

### Atomic displacement parameters (Å^2^)

**Table:**

	*U*^11^	*U*^22^	*U*^33^	*U*^12^	*U*^13^	*U*^23^
S1	0.0462 (3)	0.0734 (4)	0.0374 (3)	0.0019 (2)	−0.0064 (2)	−0.0099 (2)
O1	0.0455 (8)	0.1087 (14)	0.0376 (8)	0.0111 (8)	−0.0050 (6)	−0.0092 (8)
O2	0.0487 (9)	0.0918 (12)	0.0405 (8)	−0.0169 (8)	−0.0049 (6)	−0.0074 (8)
O3	0.0587 (10)	0.0788 (12)	0.0545 (9)	0.0018 (8)	−0.0010 (8)	0.0032 (8)
N1	0.0347 (8)	0.0593 (10)	0.0377 (8)	−0.0015 (7)	−0.0020 (6)	−0.0076 (7)
N2	0.0380 (8)	0.0664 (11)	0.0360 (8)	−0.0001 (8)	−0.0033 (6)	−0.0086 (7)
C1	0.0571 (13)	0.0664 (14)	0.0464 (11)	0.0019 (11)	−0.0066 (10)	−0.0101 (10)
C2	0.0546 (14)	0.0803 (17)	0.0663 (15)	0.0120 (12)	−0.0043 (11)	−0.0194 (13)
C3	0.0532 (13)	0.0839 (17)	0.0544 (13)	−0.0035 (12)	0.0137 (10)	−0.0200 (12)
C4	0.0623 (14)	0.0824 (17)	0.0419 (11)	−0.0071 (12)	0.0108 (10)	−0.0037 (11)
C5	0.0491 (12)	0.0660 (14)	0.0450 (11)	−0.0022 (10)	0.0038 (9)	0.0005 (10)
C6	0.0407 (10)	0.0494 (11)	0.0400 (10)	−0.0096 (8)	0.0009 (8)	−0.0072 (8)
C7	0.0434 (10)	0.0571 (12)	0.0362 (9)	−0.0061 (9)	−0.0008 (8)	−0.0039 (8)
C8	0.0406 (10)	0.0456 (10)	0.0369 (9)	−0.0042 (8)	−0.0007 (7)	−0.0043 (8)
C9	0.0415 (10)	0.0669 (14)	0.0467 (11)	0.0063 (9)	−0.0034 (9)	−0.0172 (10)
C10	0.0410 (10)	0.0857 (16)	0.0365 (10)	−0.0008 (10)	0.0006 (8)	−0.0160 (10)
C11	0.0300 (9)	0.0757 (14)	0.0398 (10)	0.0046 (9)	0.0044 (8)	−0.0075 (10)
C12	0.0768 (19)	0.0713 (18)	0.098 (2)	−0.0117 (15)	0.0022 (16)	0.0070 (16)

### Geometric parameters (Å, °)

**Table:**

S1—C8	1.6699 (19)	C3—C4	1.365 (4)
O1—C7	1.220 (2)	C3—H3A	0.9300
O2—C11	1.201 (2)	C4—C5	1.375 (3)
O3—C11	1.331 (3)	C4—H4A	0.9300
O3—C12	1.438 (3)	C5—C6	1.381 (3)
N1—C7	1.373 (2)	C5—H5A	0.9300
N1—C8	1.397 (2)	C6—C7	1.491 (3)
N1—H1A	0.8600	C9—C10	1.514 (3)
N2—C8	1.323 (2)	C9—H9A	0.9700
N2—C9	1.454 (2)	C9—H9B	0.9700
N2—H2A	0.8600	C10—C11	1.489 (3)
C1—C2	1.387 (3)	C10—H10A	0.9700
C1—C6	1.388 (3)	C10—H10B	0.9700
C1—H1B	0.9300	C12—H12A	0.9600
C2—C3	1.375 (4)	C12—H12B	0.9600
C2—H2B	0.9300	C12—H12C	0.9600
			
C11—O3—C12	116.5 (2)	O1—C7—C6	120.56 (18)
C7—N1—C8	127.78 (16)	N1—C7—C6	116.94 (17)
C7—N1—H1A	116.1	N2—C8—N1	116.04 (16)
C8—N1—H1A	116.1	N2—C8—S1	125.16 (15)
C8—N2—C9	124.28 (16)	N1—C8—S1	118.80 (14)
C8—N2—H2A	117.9	N2—C9—C10	113.98 (18)
C9—N2—H2A	117.9	N2—C9—H9A	108.8
C2—C1—C6	119.8 (2)	C10—C9—H9A	108.8
C2—C1—H1B	120.1	N2—C9—H9B	108.8
C6—C1—H1B	120.1	C10—C9—H9B	108.8
C3—C2—C1	120.0 (2)	H9A—C9—H9B	107.7
C3—C2—H2B	120.0	C11—C10—C9	114.09 (17)
C1—C2—H2B	120.0	C11—C10—H10A	108.7
C4—C3—C2	120.5 (2)	C9—C10—H10A	108.7
C4—C3—H3A	119.8	C11—C10—H10B	108.7
C2—C3—H3A	119.8	C9—C10—H10B	108.7
C3—C4—C5	119.8 (2)	H10A—C10—H10B	107.6
C3—C4—H4A	120.1	O2—C11—O3	123.6 (2)
C5—C4—H4A	120.1	O2—C11—C10	125.4 (2)
C4—C5—C6	121.0 (2)	O3—C11—C10	110.98 (18)
C4—C5—H5A	119.5	O3—C12—H12A	109.5
C6—C5—H5A	119.5	O3—C12—H12B	109.5
C5—C6—C1	118.93 (19)	H12A—C12—H12B	109.5
C5—C6—C7	117.30 (18)	O3—C12—H12C	109.5
C1—C6—C7	123.63 (18)	H12A—C12—H12C	109.5
O1—C7—N1	122.51 (18)	H12B—C12—H12C	109.5

### Hydrogen-bond geometry (Å, °)

**Table:**

*D*—H···*A*	*D*—H	H···*A*	*D*···*A*	*D*—H···*A*
N2—H2A···O1	0.86	1.94	2.625 (2)	136
N1—H1A···O2^i^	0.86	2.17	3.022 (2)	169
C1—H1B···O2^i^	0.93	2.50	3.187 (3)	130
C9—H9A···O1^ii^	0.97	2.59	3.464 (3)	150

Symmetry codes:  (i) *x*+1, *y*, *z*; (ii) −*x*, −*y*+1, −*z*+1.
